# Influence of marital status on overall survival in patients with ovarian serous carcinoma: finding from the surveillance epidemiology and end results (SEER) database

**DOI:** 10.1186/s13048-019-0600-7

**Published:** 2019-12-30

**Authors:** Pei Luo, Jian-Guo Zhou, Su-Han Jin, Ming-Song Qing, Hu Ma

**Affiliations:** 1 Department of Oncology, The Second Affiliated Hospital of Zunyi Medical University, Zunyi, 563000 China; 2 Department of Radiation Oncology, Universitätsklinikum Erlangen, Erlangen, 91054 Germany; 3 Department of Orthodontics, Affiliated Stomatology Hospital of Zunyi Medical University, Zunyi, 563000 China; 4 Department of Orthopedics, Affiliated Hospital of Zunyi Medical University, Zunyi, 563000 China

**Keywords:** Marital status, SEER, Ovarian serous carcinoma, Overall survival

## Abstract

**Objective:**

This study is to investigate the relationship between marital status and prognosis of patients with ovarian serous carcinoma.

**Results:**

We performed data analysis from 19,276 patients identified from the SEER database of the National Cancer Center of the United States. 57.8% of the patients were married, 13.0% unmarried, and 29.2% separated/ divorced/widowed (SDW). The median overall survival time ofthe unmarried group and the married group are 48 months and 52 months respectively. Univariate Cox regression analysis showed that the patients with serous ovarian cancer in the unmarried group resulted in a hazard ratio (HR) of 1.14 (95% CI: 1.08–1.19%; *P* < 0.001), comparing to SDW group with a HR of 1.02 (95% CI: 0.98–1.19%; *P* = 0.26). However, the SDW group was not statistically significantly different from the married group. (median 32 vs 52 months). Multivariate Cox regression analysis presented the unmarried group leading to a HR of 1.05 (95% CI: 1.00–1.11%; *P* = 0.05), and the SDW group was not significant with a HR of 0.99 (95% CI: 0.95–1.03%; *P* = 0.57).

**Conclusion:**

Unmarried patients with ovarian serous carcinoma have higherHRof overall survival. After controlling age, race, grade, radiation and year of diagnosis, unmarried patients were found to have a significantly higher risk of OS. Consequently, these patients are suggested to obtain more focused healthcare for the management of ovarian serous carcinoma.

## Background

Ovarian cancer is one of the most common malignant tumors in the female reproductive system. Although the incidence of ovarian cancer is lower than that of cervical cancer and uterine cancer, the mortality rate of ovarian cancer is the highest in gynecological malignancies [1–3]. The early clinical symptoms are usually concealed, and there are no effective methods for early diagnosis. The disease progresses rapidly, thus more than two-thirds of patients with ovarian cancer were diagnosed as advanced stage which is too late for radical surgery. In addition, chemotherapy resistance is one of the main factors leading to high mortality in ovarian cancer. Many studies have confirmed that marital status may affect the prognosis of various tumor types, including prostate cancer [4], renal carcinoma [5], colorectal cancer [6, 7], and mouth cancer [8] etc. It is un-known how marital status affects the cancer prognosis so far. Studies have shown that clinical decision-making is influenced by many factors, including marital status. Different clinical decisions may lead to different prognosis [9]. However, there is no literature reporting the impact of marital status on the prognosis of ovarian cancer. This study investigated the relationship between marital status and serous ovarian cancer prognosis by data analysis of the SEER database of the National Cancer Center of the United States.

## Material and methods

### Patient selection

The American Surveillance, Epidemiology, and End Results (SEER) database was used to acquire data of patients who were diagnosed with ovarian serous carcinoma. The data was analyzed by SEER*STAT 8.3.2, and ovarian cancer patients were filtrated from year 1973 to year 2013 by diagnosed date.

*Patient inclusion criteria:*

(i) Patient was histologically confirmed with serous ovarian cancer and there was presence of only one primary tumor
(ii) Patient’s age was recorded over 18 years old
(iii) Patient’ race was recorded
(iv) Patient’ marriage status was logged
(v) Patient’s histological grade was stated
(vi) There was active follow-up

*Patient exclusion criteria:*

(i) Patient’s age was unknown
(ii) Patient’s race was not recorded
(iii) Patient’s marriage status was not stated
(iv) There was no complete histological grade information
(v) The cause of death was unknown
(vi) Patient’s survival period was not recorded

Finally, there were 19,276 patients meeting all the inclusion criteria and were included in the study. There was no staging data for failing to get the FIGO stage.

### Methodology

This retrospective study was accorded to the marital status, the patients were divided into the married group, unmarried group and SDW group (divorced, separated, widowed), the outcome index was overall survival (OS).

### Statistical analysis

The main endpoint of this study is the overall survival (OS) extracted from the SEER database. Data was extracted and processed by Perl 5.26.2 software, and further processed by R 3.35 software and its related software packages. Chi-square test was used for statistical analysis of clinical frequency data, univariate analysis and survival curve was derived from Kaplan-Meier estimation. Additionally, multivariate Cox proportional hazard regression model was used to evaluate the mortality risk. All statistical tests were double-sided, and *P* ≤ 0.05 was considered as statistically significant.

## Results

### Relationship between marital status and clinic-pathological characteristics

A total of 19,276 patients aged from 19 to 100 were included in the study, with an average age of (62.98 ± 13.75) years old. Among all the patients, 1105 were black, 17,084 were white, 1087 were other races. There were 1450, 4445, 10,225, 3156 cases of histological grade I, II, III, IV, respectively, as shown in Table 1.

**Table 1:** basic characteristics of patients with serous ovarian cancer (*n* = 19,276)

Item	n	Marital status			*P* value
		married	single	SDW	
Total number	19,276	11,130	2520	5626	
Race					< 0.0001
Black	1105	415	265	425	
White	17,084	10,027	2111	4946	
Other	1087	688	144	255	
Age/years					< 0.0001
1–39	929	513	310	106	
40–49	2674	1737	556	381	
50–59	4877	3170	746	961	
60–69	5292	3286	529	1477	
70–79	4047	2017	280	1750	
80+	1457	407	99	951	
Diagnostic year					< 0.0001
1973–1999	7067	4099	764	2204	
2000–2005	6269	3627	819	1823	
2006–2010	4225	2426	631	1168	
2011+	1715	978	306	431	
Histological grading					0.0497
I	1450	886	168	396	
II	4445	2587	577	1281	
III	10,225	5834	1388	3003	
IV	3156	1823	387	946	
Radiotherapy					0.1783
Yes	18,822	10,886	2451	5485	
No	454	244	69	141	

Among all the patients, there were 11,130 patients (57.8%) in the married group, 2520 (13%) in the unmarried group, and 5626 (29.2%) in SDW group. The overall age of the unmarried group was relatively young, with the median age of 54 years old and the average age of (54.85 ± 13.85) years old. The married group has a median age of 60 and mean age of (59.61 ± 11.79). The SDW group hasmedian age of 69 and mean of (66.75 ± 12.50). The difference of age among groups was statistically significant (*P* < 0.001). In the years of treatment, there was a significant difference between the married group and the other two groups (*P* < 0.001) from year 1973 to year 1999. The proportion of grade III in the SDW group was the highest, and it was significantly different between the married group and the other two groups (*P* = 0.0497). There was no significant difference in the distribution of radiotherapy among the three groups (*P* = 0.1783).

### Relationship between marital status and prognosis

**Fig. 1:**
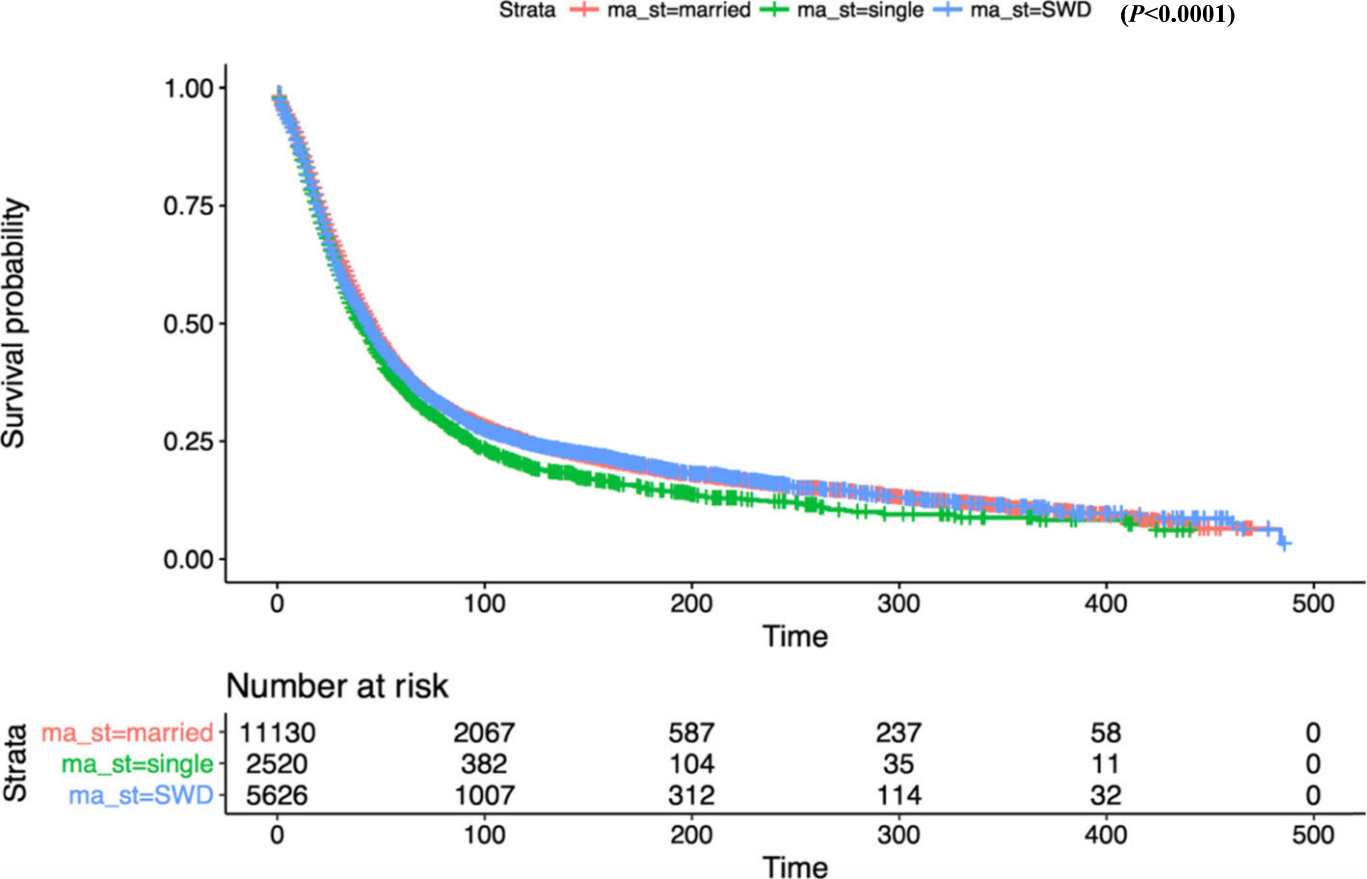
Kaplan-Meier survival curves of overall survival by married status

In the entire cohort, the median overall survival time was demonstrated significantly shorter in the unmarried group comparing with the married group (median 48 vs 52 months). Univariate Cox regression analysis showed that marital status was associated with overall survival. The serous ovarian cancer patients in unmarried group had a HR of 1.14 (95% CI: 1.08–1.19%; *P* < 0.001). However, the median overall survival in SDW group was numerically but not statistically significantly different from the married group (median 32 vs 52 months), with a HR of 1.02 (95% CI: 0.98–1.19%; *P* = 0.26). Further calculation revealed multivariate COX regression analysis presented the unmarried group leading to a HR of 1.05 (95% CI: 1.00–1.11%; *P* = 0.05). Thus the SDW group was no longer statistically significant with a HR of 0.99 (95% CI: 0.95–1.03%; *P* = 0.57) (Fig. 1).

**Fig. 2:**
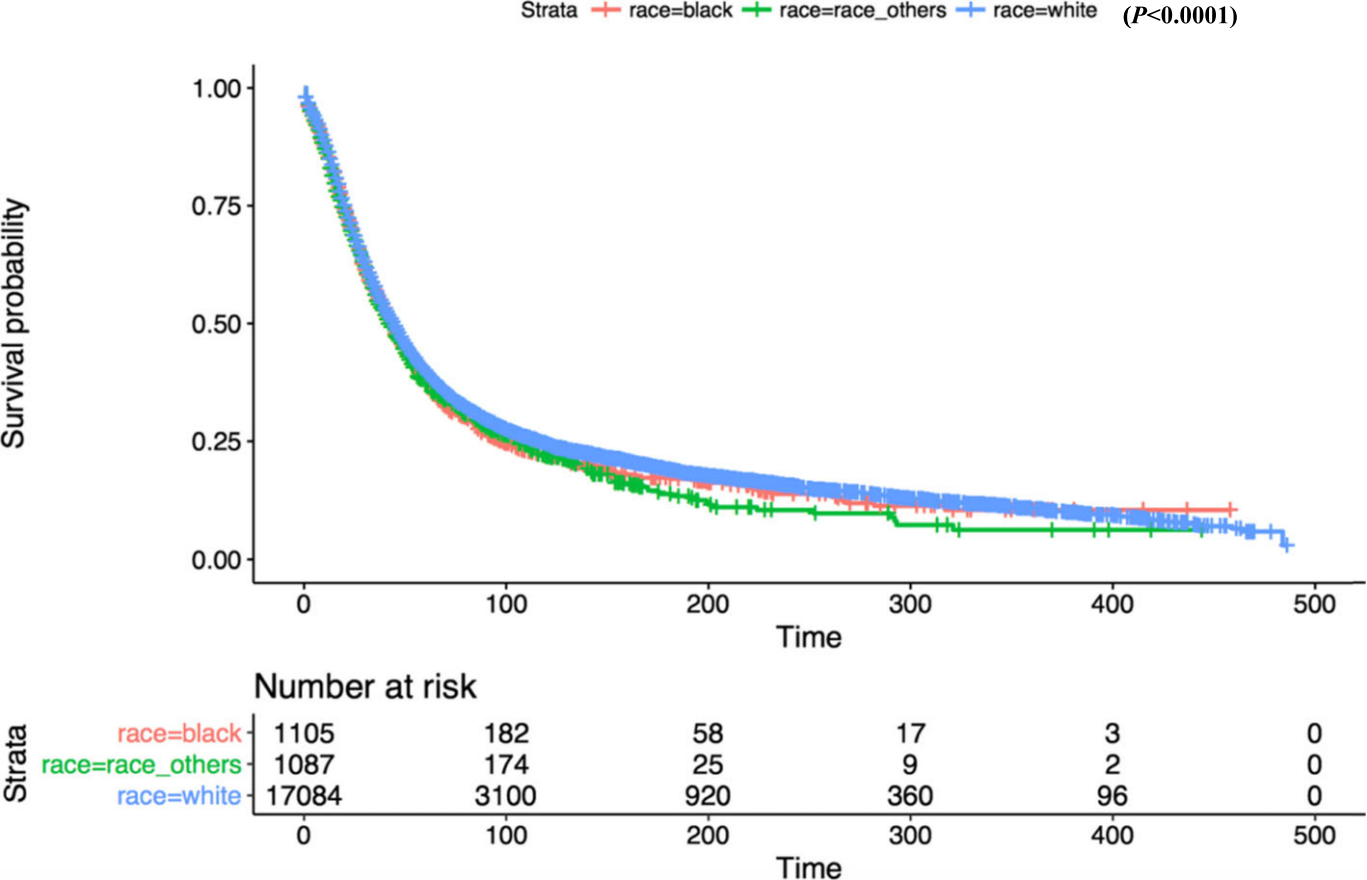
Kaplan-Meier survival curves of overall survival by race

**Fig. 3:**
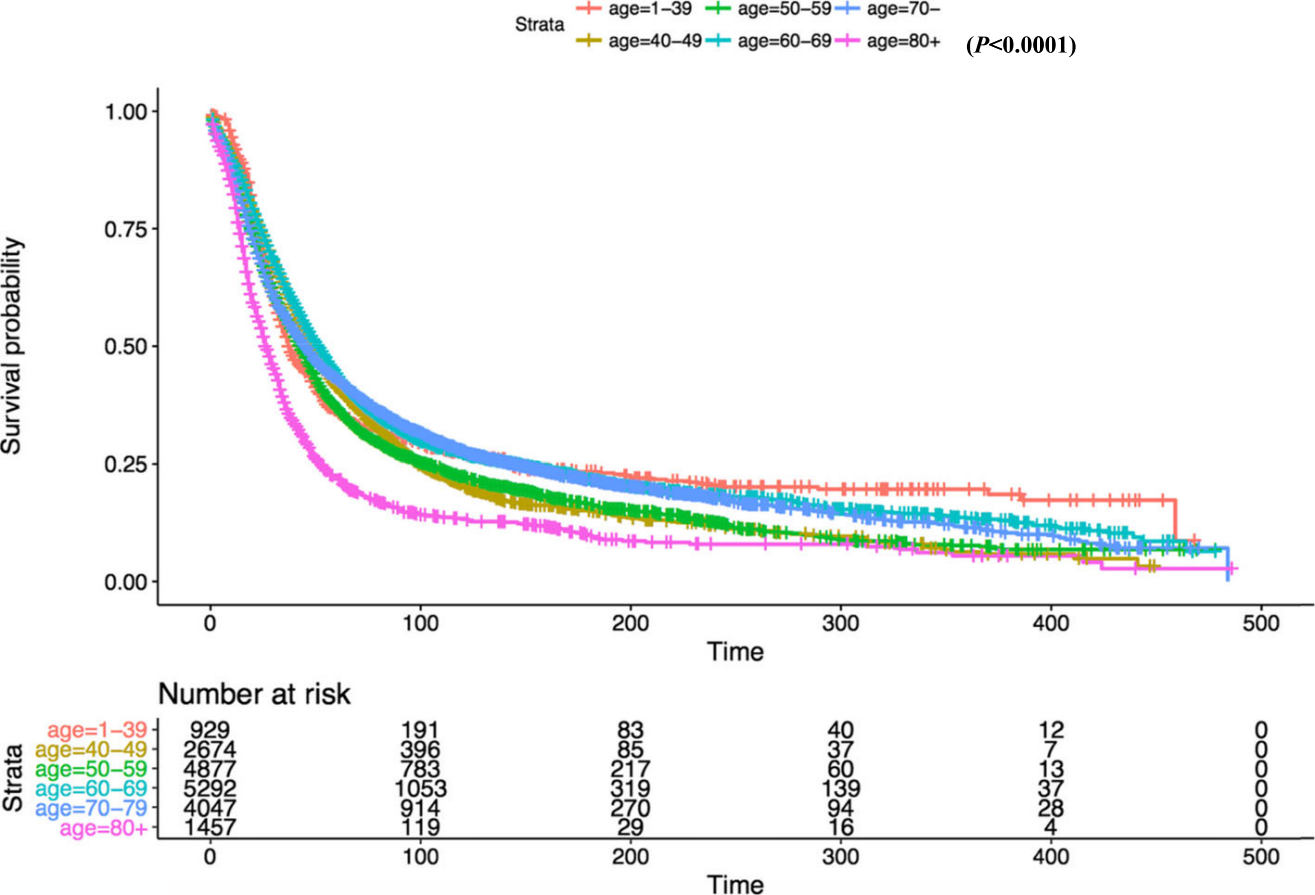
Kaplan-Meier survival curves of overall survival by age

**Fig. 4:**
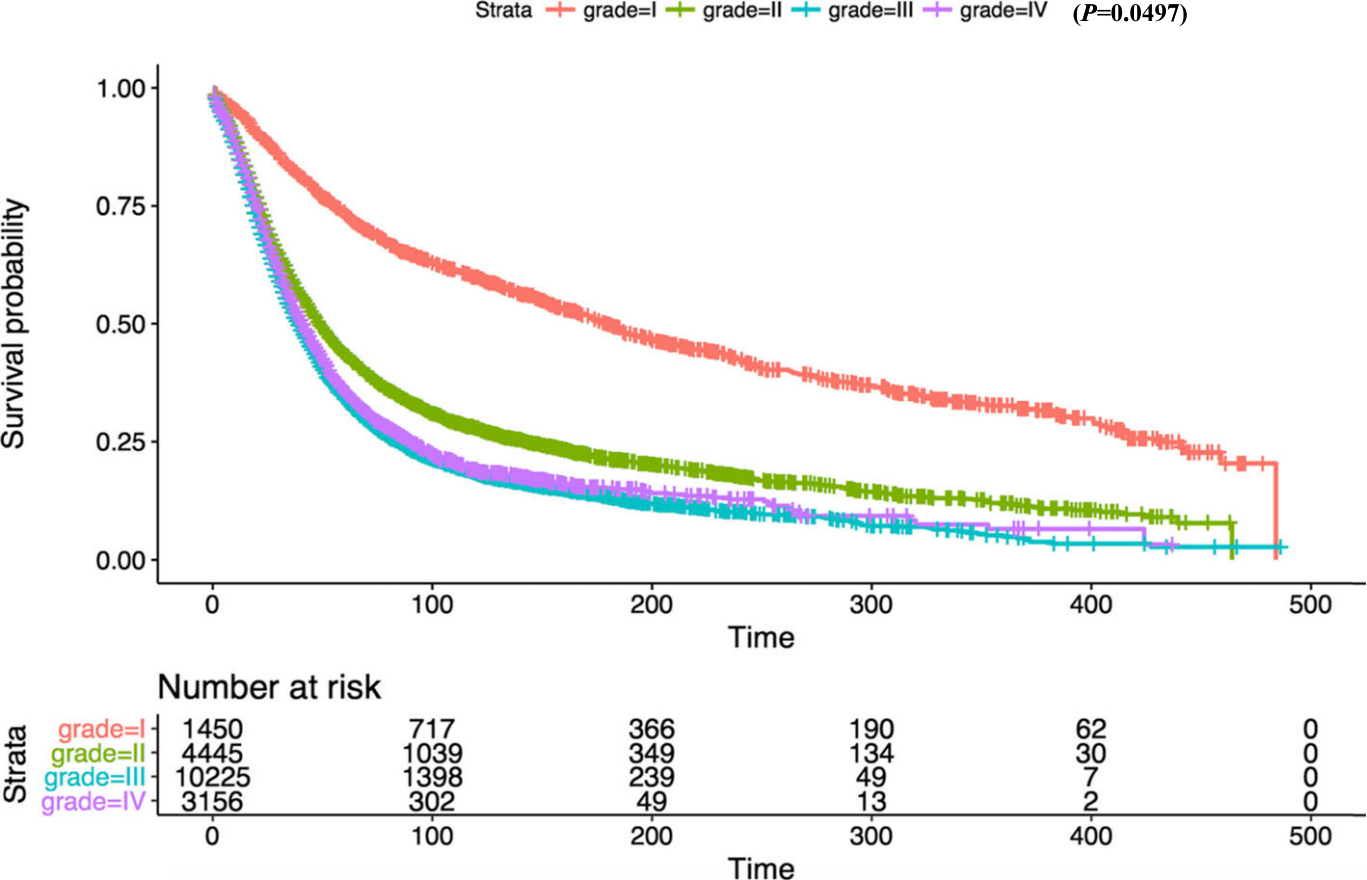
Kaplan-Meier survival curves of overall survival by grade

**Fig. 5:**
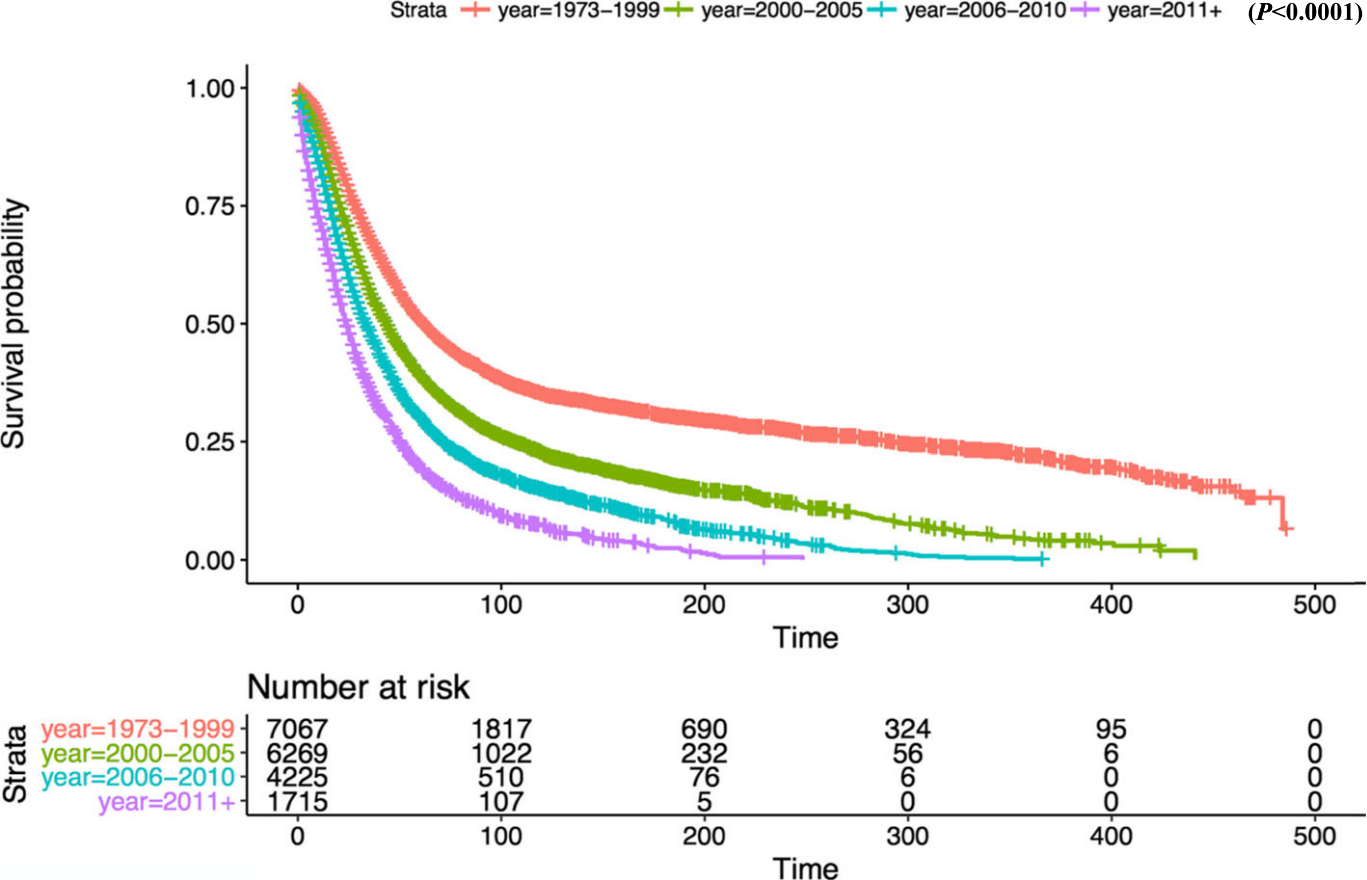
Kaplan-Meier survival curves of overall survival by year

**Fig. 6:**
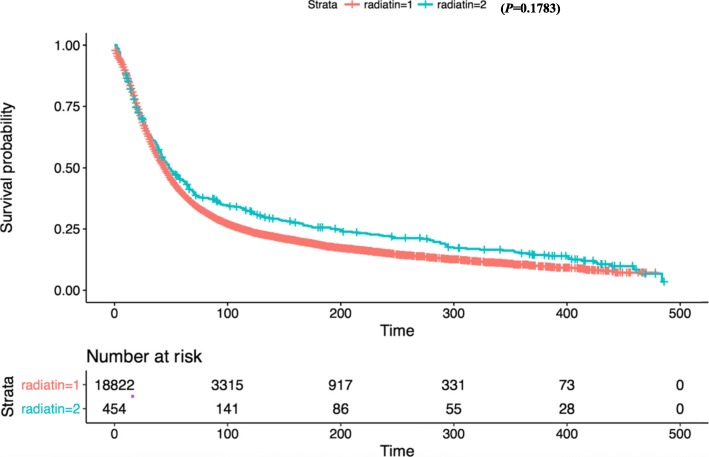
Kaplan-Meier survival curves of overall survival by radiation

Kaplan-Meier survival analysis revealed that there was no significant difference in OS between white and other races (Fig. 2). In terms of patient age, OS was significantly reduced in the 80+ year old group compared with the 1–39 age group (Fig. 3). OS in grade II-IV group was significantly lower than that in grade I group in histological type (Fig. 4). In terms of treatment years, OS in different years of treatment was significantly different from that in 1973–1999 (Fig. 5). Patients who received radiation therapy had a better prognosis than those who did not (Fig. 6). The specific single factor and multifactor results are shown in Table 2.

**Table 2:** Univariate and multivariable Cox regression model of overall survival of patients with Ovarian Serous Carcinoma

Case	Univariate		Multivariable	
	HR(95%CI)	*P* value	HR(95%CI)	*P* value
Marital status				
Ref = married				
Single	1.14 (1.08–1.19)	< 0.001	1.05 (1.00–1.11)	0.05
SDW	1.02 (0.98–1.06)	0.264	0.99 (0.95–1.03)	0.565
Race				
Ref = Black				
White	0.95 (0.88–1.02)	0.128	1.01 (0.94–1.09)	0.709
Other	1.03 (0.93–1.13)	0.593	1.03 (0.93–1.13)	0.578
Age				
ref. = (1–39)				
40–49	1.07 (0.98–1.17)	0.151	0.88 (0.80–0.96)	0.005
50–59	1.15 (1.05–1.25)	0.001	0.97 (0.89–1.05)	0.446
60–69	0.95 (0.87–1.03)	0.232	0.86 (0.79–0.94)	0.001
70–79	1.02 (0.93–1.11)	0.725	0.89 (0.81–0.97)	0.007
80+	1.71 (1.55–1.88)	< 0.001	1.50 (1.35–1.65)	< 0.001
Histological types				
Ref = I				
II	2.24 (2.07–2.43)	< 0.001	2.11 (1.94–2.28)	< 0.001
III	2.93 (2.71–3.16)	< 0.001	2.66 (2.47–2.88)	< 0.001
IV	2.75 (2.52–2.99)	< 0.001	2.49 (2.28–2.71)	< 0.001
Diagnostic year				
Ref = 1973–1999				
2000–2005	1.50 (1.43–1.56)	< 0.001	1.40 (1.34–1.46)	< 0.001
2006–2010	1.99 (1.90–2.08)	< 0.001	1.83 (1.75–1.92)	< 0.001
2011+	2.80 (2.64–2.97)	< 0.001	2.72 (2.56–2.89)	< 0.001
Radiotherapy				
Ref = No				
Yes	0.86 (0.77–0.95)	0.004	1.06 (0.95–1.17)	0.313

## Discussion

A great number of research focused on the influence of marital status on the prognosis of cancer patients. Mehul K et al. [10] reported that women with invasive cervical cancer demonstrated a significant effect of marital status on survival, Married women tended to have more favorable prognostic and treatment-related characteristics compared with single, divorced/separated, and widowed women. A study by Wang X, et al. [11] found that unmarried patients, including divorced/separated, widowed and never married were at significantly greater risk of mortality after diagnosis of epithelial ovarian cancer. Osborne C et al. [12] reported that older married women had a lower mortality rate after being diagnosed with breast cancer, while unmarried women had an increased risk of dying from breast cancer. Those studies suggested that marital status is associated with the prognosis of cancer patients. In different studies, the effect of marital status on the mortality rate of tumor patients may be an independent factor. Marital status may also interact with tumor stages and the selection of treatment options, resulting in a significant impact on the survival rate of tumor patients.

Our study was the first to analyze the relationship between marital status and prognosis in patients with serous ovarian cancer. The results of this study showed that serous ovarian cancer patients with good marital status had the better prognosis and lower risk of death, compared with single and SDW (divorced, separated, widowed) women. We speculate that this result is due to the fact that marital status is considered to be the most effective social support, mainly from the spouse. The main treatment method of ovarian cancer patients is by surgery. The surgery methods according to the FIGO staging of ovarian cancer are divided into complete staging operation and staged operation with fertility preservation. Complete staging of the operation includes a bilateral appendix, uterus, omentum excision, and pelvic and retroperitoneal lymphadenectomy. For advanced patients with extensive pelvic metastasis, cytoreductive surgery is recommended as far as possible. Surgical decisions are influenced by many factors including marital status [13].

A great many of research studies have been confirmed that cancer patients with harmonious marriage have a better prognosis. This includes patients diagnosed with oral cancer, prostate cancer, kidney cancer, colorectal cancer, gynecological tumors and other tumors [4–6, 8, 14]. Cancer patients with harmonious marriage have a better prognosis because the cancer stage is earlier and better than that of patients with unhealthy marriage. And these patients are accompanied by their spouses, with mental and economic support, which can be integrated into clinical decision-making. Women are vulnerable to emotional factors, spouses can provide patients with timely and effective emotional and social support environment, and accompany patients to face the pressure of cancer. Studies performed by Smits S, et al. [15] have shown that cancer risk components in perceived threat are a unique predictor of ovarian cancer risk factors. Among women at increased risk, the fear component of perceived threat may be more influential than susceptibility in influencing early performance behavior, thus targeted interventions are needed to minimize cancer-related concerns in this population. This highlights the close relationship between psychological state and the incidence of ovarian cancer. This suggests that a variety of interventions can be combined to improve the mental state and prognosis of patients.

In this study, it was found that unmarried patients with serous ovarian cancer had poorer overall survival than married patients. Other factors including race, age, length of service, histological grade, and radiation therapy were used to verify the robustness of the Cox multivariate analysis. However, in the analysis of marital status’ impact on OS, it was found the prognosis of SDW patients was not worse than married patients. This phenomenon may vary according to race and histological grading of disease. It may be also related to insufficient research sample size and insufficient statistical test efficacy.

This analysis showed that unmarried patients were higher than the other two groups. The risk is independent of baseline factors which may affect patients’ willingness to seek medical treatment. Therefore, unmarried patients had poor prognosis that may be caused by many factors. This is important for medical staff to provide more attention and concern in the diagnosis and treatment of those patients. There are several limitations in our study. First of all, this is a retrospective study, which has a greater bias probability comparing to the prospective study. Secondly, the SEER database provided only baseline marital status and did not further study the relationship between marital status dynamics and tumor prognosis. Third, the database did not provide FIGO staging and surgery, chemotherapy and other important factors affecting patients’ prognosis [1–3, 16, 17] that may lead to bias. However, this study is the first to examine the impact of marital status on the prognosis of ovarian serous carcinoma based on big data.

## Conclusion

Our study showed that marital status plays a significant role in the prognosis of patients with ovarian serous carcinoma. Married patients had longer survival time than unmarried including divorced/separated, widowed and never married patients. From a social point of view, marriage plays as the main source of social support. Peer support programs could positively influence patient expectation and coping with diagnosis and treatment [18], such that it can directly lead to better prognosis [19]. This can also reduce the probability of depression and anxiety [20]. Therefore, the community and clinicians should provide more care and support to unmarried patients, including divorced/separated, widowed and never married patients.

## Data Availability

The dataset generated and analyzed during the current study is available in the Surveillance Epidemiology and End Results (SEER) Database repository [https://seer.cancer.gov/].

## Abbreviations

FIGO: Federation international of gynecology and obstetrics
HR: Hazard ratio
OS: Overall survival
SDW: Divorced, separated, widowed
SEER: Surveillance epidemiology and end Results
